# Emerging role of non-coding RNAs in neuroinflammation mediated by microglia and astrocytes

**DOI:** 10.1186/s12974-023-02856-0

**Published:** 2023-07-23

**Authors:** Ruicheng Yang, Bo Yang, Wei Liu, Chen Tan, Huanchun Chen, Xiangru Wang

**Affiliations:** 1 National Key Laboratory of Agricultural Microbiology, College of Veterinary Medicine, Huazhong Agricultural University, Wuhan, 430070 China; 2 Key Laboratory of Preventive Veterinary Medicine in Hubei Province, The Cooperative Innovation Center for Sustainable Pig Production, Wuhan, 430070 China; 3 Frontiers Science Center for Animal Breeding and Sustainable Production, Wuhan, 430070 China; 4 Wuhan Keqian Biological Co., Ltd., Wuhan, 430070 China; 5 Wuhan Academy of Agricultural Sciences, Wuhan, 430070 China

**Keywords:** Neuroinflammation, miRNAs, lncRNAs, circRNAs, Microglia, Astrocytes

## Abstract

Neuroinflammation has been implicated in the initiation and progression of several central nervous system (CNS) disorders, including Alzheimer’s disease, Parkinson’s disease, amyotrophic lateral sclerosis, multiple sclerosis, ischemic stroke, traumatic brain injury, spinal cord injury, viral encephalitis, and bacterial encephalitis. Microglia and astrocytes are essential in neural development, maintenance of synaptic connections, and homeostasis in a healthy brain. The activation of astrocytes and microglia is a defense mechanism of the brain against damaged tissues and harmful pathogens. However, their activation triggers neuroinflammation, which can exacerbate or induce CNS injury. Non-coding RNAs (ncRNAs) are functional RNA molecules that lack coding capabilities but can actively regulate mRNA expression and function through various mechanisms. ncRNAs are highly expressed in astrocytes and microglia and are potential mediators of neuroinflammation. We reviewed the recent research progress on the role of miRNAs, lncRNAs, and circRNAs in regulating neuroinflammation in various CNS diseases. Understanding how these ncRNAs affect neuroinflammation will provide important therapeutic insights for preventing and managing CNS dysfunction.

## Introduction

The central nervous system (CNS) is considered an immune-privileged site compared to the peripheral tissues [1]. The lack of resident dendritic cells and the relatively anti-inflammatory environment of the neural tissue result in a muted innate immune response within the CNS parenchyma [2]. However, the immune response in the CNS is widespread, and CNS homeostasis is highly dependent on the balance of the innate immune response. Neuroinflammation is a complex innate immune response involving reactive CNS elements that alter homeostasis. Inflammation is mediated by proinflammatory cytokines, chemokines, reactive oxygen species, and secondary messengers. Initially, neuroinflammation plays a beneficial role by eliminating microbes or promoting tissue repair. Conversely, uncontrolled neuroinflammation can become detrimental and cause pathogenic tissue damage [3]. Therefore, understanding the cellular and molecular regulators of neuroinflammation may provide helpful clues for developing new therapeutic interventions for treating CNS diseases.

Glial cells are critical components of the CNS and include microglia, astrocytes, and oligodendrocytes [4]. Microglia are the resident phagocytes of the innate immune system and the most motile cells in the CNS that regulate brain development, maintain neuronal networks, and modulate CNS injury and infection [5]. Microglia are activated earlier than other glial cells and are first responders to various CNS insults. Microglial activation is accompanied by morphological changes and is categorized into two opposing types: M1 and M2 phenotypes [6]. M1 microglia exhibit proinflammatory and neurotoxic states and are involved in the acute defense against pathogenic organisms, whereas M2 microglia are involved in the resolution of inflammation and tissue repair. Astrocytes are the most abundant type of glial cells in the CNS and are essential for brain homeostasis. Astrocytes provide an energy substrate for neurons, maintain the extracellular balance of ions and fluid, and contribute to the formation and maintenance of the blood–brain barrier (BBB) [7]. In addition, astrocytes, which are immune-competent cells within the brain, are critical regulators of innate and adaptive immune responses in the injured CNS. Upon CNS insult, astrocytes undergo proliferation and morphological changes, termed astrogliosis [8]. Increased expression of the glial fibrillary acidic protein (GFAP) is a marker of astrogliosis. Astrocytes can exacerbate inflammatory responses and aggravate tissue damage, but they can also promote immunosuppression and tissue repair. Thus, the specific role of glial cells depends on their unique characteristics and the nature of stimuli present in the inflammatory environment [9].

High-throughput sequencing techniques have revealed that only 1–2% of the human genome encodes proteins, but up to 90% of genome-produced transcripts have no protein-coding capacity and are referred to as non-coding RNAs (ncRNAs) [10]. ncRNAs are divided into two subclasses based on their biological functions: housekeeping and regulatory ncRNAs. Housekeeping ncRNAs (tRNA, rRNA, etc.) are constitutively and ubiquitously expressed, and essential for cell maintenance [11]. The three distinct classes of regulatory ncRNAs are microRNAs (miRNAs), long non-coding RNAs (lncRNAs), and circular RNAs (circRNAs) [12]. miRNAs are a large family of short, single-stranded ncRNAs, approximately 22 nucleotides (nt) in length. They play an important role in post-transcriptional gene regulation by targeting 3ʹ-untranslated regions (UTRs), resulting in translational repression or degradation of their messenger RNA (mRNA) targets [13]. Studies indicate that miRNAs are also capable of translation promotion and DNA binding for repression or activity [14, 15]. Importantly, at least 60% of protein-coding genes are targeted by miRNAs, leading to their broad regulatory roles in diverse biological processes [16]. lncRNAs are defined as transcripts longer than 200 nt without coding capacity. Based on their genomic locations, lncRNAs are classified into four different types: long intergenicncRNAs, antisense, intronic, and bidirectional lncRNAs [17]. In contrast to miRNAs, lncRNAs have been proposed to have regulatory functions in gene expression at both the transcriptional and post-transcriptional levels in various cellular and biological processes [18]. They regulate the expression of genes located on the same chromosome (acting in cis) or genes from different chromosomes (acting in trans) by interacting with proteins, DNAs, or RNAs. circRNAs are covalently bound endogenous molecules generated by a non-canonical splicing event called back-splicing [19]. During back-splicing, a downstream splice donor site is linked to an upstream splice acceptor site, and a phosphodiester bond at the junction site ligates the RNA cycle [20]. circRNAs are molecules with high stability due to the absence of 5ʹ caps and 3ʹ poly-A tails, and the covalently closed ring structure protects against exonuclease-mediated degradation [21]. In addition, circRNAs exhibit cell- and tissue-specific patterns and are particularly abundant in the human brain [22]. Recent investigations have shown that circRNAs are involved in neuronal function, cell proliferation, and innate immunity. At the molecular level, circRNAs modulate gene expression by sponging miRNAs, interacting with proteins, and regulating transcription and splicing [23].ncRNAs are differentially expressed in various neurological disorders associated with neuroinflammation, including chronic neurodegenerative diseases, acute neurodegenerative diseases, and CNS infectious diseases. In addition, microglia and astrocytes are closely associated with the outcome and progression of CNS disorders. The emerging links between ncRNAs, glial cells, and CNS disorders have opened a new field of diagnostic and therapeutic opportunities. In this review, we summarize the current research on the role of ncRNAs in microglial- and astrocyte-mediated neuroinflammation in CNS pathologies.

### Alzheimer’s disease

Alzheimer’s disease (AD) is one of the most common age-related neurodegenerative diseases, characterized by memory loss, cognitive impairment, and various neuropsychiatric disorders [24]. The neuropathological hallmarks of AD include the deposition of amyloid beta (Aβ) peptide and intracellular neurofibrillary tangles. Aβ plaques are formed by the cleaved products of the amyloid precursor protein, and neurofibrillary tangles are composed of hyperphosphorylated forms of the microtubule-associated protein tau [25]. Microglia and astrocytes play important roles in mediating neuroinflammation in AD brain tissue. Microglia change their morphology from a ramified to an amoeboid state, and astrocytes show reactive astrogliosis during this disease [26].

Persistent activation of microglia and astrocytes can trigger inflammatory responses, leading to neuronal damage, and ultimately AD [27]. miRNAs play important regulatory roles in glial cell-mediated neuroinflammation in AD. For example, treatment with miR-22-loaded exosomes significantly inhibited microglial activation and inflammatory factor expression by inhibiting pyroptosis [28]. Exosomal delivery of miR-146a suppresses astrocyte inflammation by targeting TRAF6, which promotes synaptogenesis and ameliorates cognitive impairment [29]. In a 3 × Tg AD animal model, miR-155 was significantly upregulated in the mouse brain and Aβ-activated microglia and astrocyte cultures. miR-155 contributes to the production of interleukin 6 (IL-6) and Interferon-β (IFN-β) by sponging off suppressor of cytokine signaling 1 (SOCS1) [30]. It has been reported that cerebrospinal fluid from individuals with AD contains elevated levels of let-7b, and extracellular introduction of let-7b into the cerebrospinal fluid of mice by intrathecal injection resulted in neurodegeneration. Mechanistically, let-7b can activate microglia by acting as a damage-associated molecular pattern against toll-like receptor 7 (TLR7) [31]. In an AD rat model, inhibition of miR-592 attenuated oxidative stress injury in astrocytes by upregulating KIAA0319, thereby alleviating neuronal damage [32]. In another study, miR-135 blockade in astrocytes inhibited neuronal apoptosis and promoted neurite outgrowth by targeting thrombospondin 1 [33]. Aβ aggregation is a major pathogenic factor in AD, and emerging evidence suggests that miRNAs may modulate Aβ production. It was reported that miR-206 expression was significantly upregulated in AD patients, and miR-206 induced inflammation and Aβ release in microglia by targeting insulin-like growth factor 1 (IGF1) [34]. Triggering receptor expressed on myeloid cell 2 (TREM2) is an immunoreceptor primarily found on microglia in the CNS and is critical for Aβ42 peptide clearance. However, miR-34a inhibits TREM2 expression and attenuates the ability of microglia to clear self-aggregating Aβ42 peptides [35]. Moreover, targeted modulation of miR-155 expression regulates the ability of microglia to catabolize fibrillar Aβ1-42; overexpression of miR-155 decreases fibrillar Aβ1-42 catabolism, whereas deletion of miR-155 promotes fibrillar Aβ1-42 catabolism [36]. In addition, the expression of miR-331-3p and miR-9-5p increased in a late-stage AD mouse model. Overexpression of miR-331-3p and miR-9-5p impairs autophagic activity and promotes Aβ formation, whereas their inhibition reduces microglial activation, enhances Aβ clearance, and improves cognition via autophagy activation. Mechanistically, miR-331-3p targets the autophagy receptor sequestosome 1, and miR-9-5p targets the autophagy receptor optineurin [37]. Another study found that miR-138 upregulation in vivo caused an increase in endogenous Aβ42 production as well as changes in inflammatory markers by targeting Sirt1 [38].

**Fig. 1:**
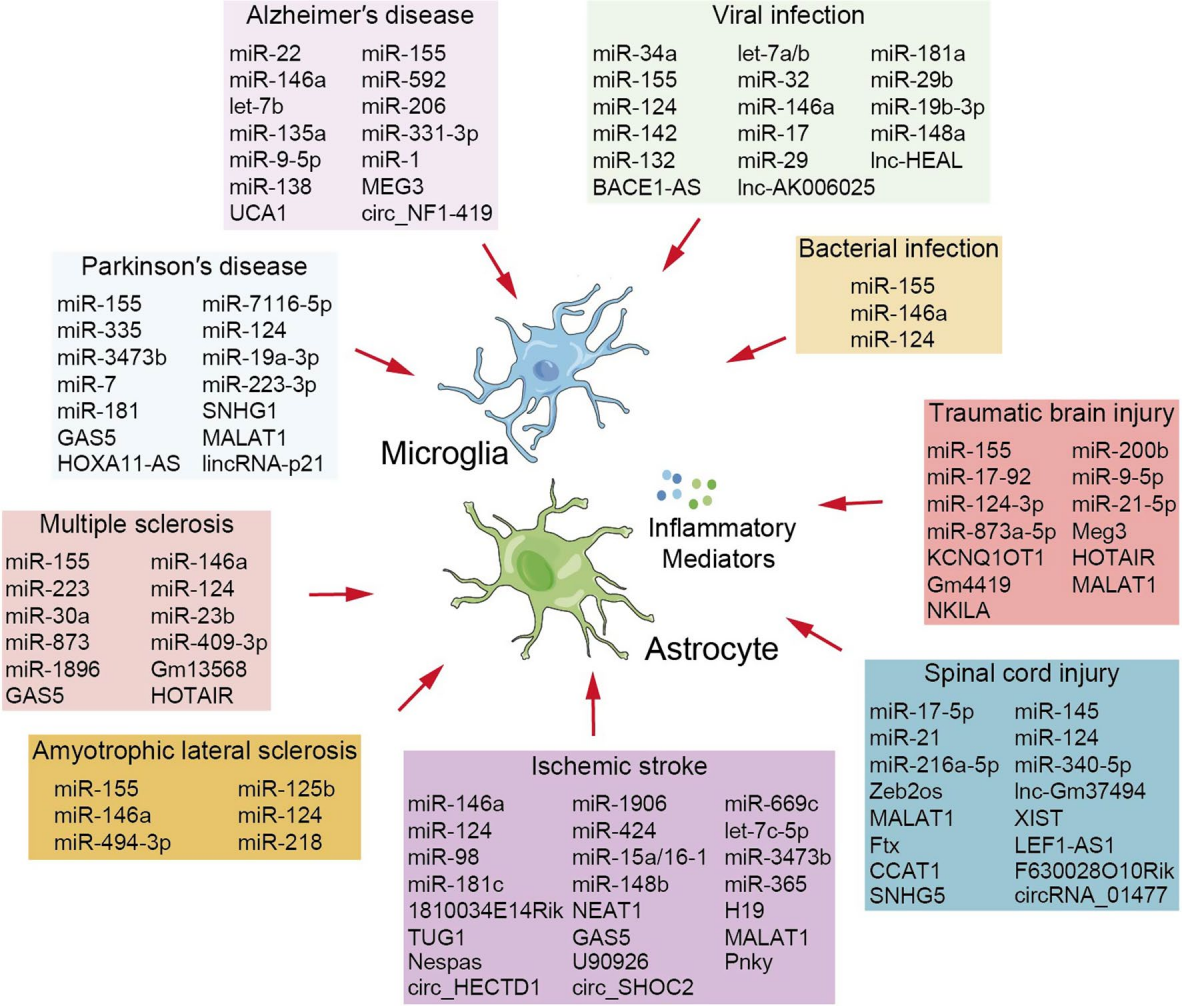
Brief summary of miRNAs, lncRNAs and circRNAs as a factor controlling neuroinflammation

In addition to miRNAs, lncRNAs and circRNAs play important regulatory roles in glial cell-mediated neuroinflammation in AD. For example, the expression of lncRNA maternally expressed gene 3 (lncMEG3) decreased in the tissues of AD rats, whereas overexpression of lncMEG3 inhibited astrocyte activation, suppressed inflammatory injury, and alleviated neuronal damage [39]. Neural stem cells (NSCs) can self-renew and generate glial and neuronal lineages. lncRNA urothelial carcinoma-associated 1 (lncUCA1) is upregulated in NSCs and can modulate NSC differentiation. Inhibition of lncUCA1 promotes NSC differentiation into neurons and suppresses its differentiation into astrocytes. Mechanistically, lncUCA1 binds to miR-1 and regulates NSC proliferation and differentiation by targeting Hes1 [40]. In a senescent cell aging model, 7376 circRNAs were identified in primary astrocytes cultured in d-galactose. Among them, CircNF1-419 was significantly upregulated and involved in the modulation of astrocyte autophagy. It enhanced autophagy by binding to dynamin-1 and adaptor protein 2 B1 (AP2B1) [41] (Fig. 1, Table 1).

**Table 1:** ncRNAs in the regulation of astrocyte- and microglia-mediated neuroinflammation in chronic neurodegenerative diseases

	ncRNAs	Target	Function
AD	miR-22↑		Improve neurological function and neuroinflammation [28]
AD	miR-146a↑	TRAF6	Led to synaptogenesis and correction of cognitive impairment [29]
AD	miR-155↑	SOCS1	Contributes to neuroinflammation [30]
AD	let-7b↑	TLR7	Promotes microglia activation [31]
AD	miR-592↑	KIAA0319	Promotes oxidative stress injury [32]
AD	miR-135a↑	Thrombospondin 1	Promotes neuronal apoptosis [33]
AD	miR-206↑	IGF1	Induces inflammation and Aβ release in microglia [34]
AD	miR-34a↓	TREM2	Inhibits the ability of microglia to catabolize Aβ42 [35]
AD	miR-155↑		Promotes the ability of microglia to catabolize Aβ42 [36]
AD	miR-331-3p	Sequestosome 1	Ameliorates Alzheimer's disease by enhancing autophagy [37]
AD	miR-9-5p	Optineurin	Ameliorates Alzheimer's disease by enhancing autophagy [37]
AD	miR-138↑	Sirt1	Contributes to Aβ42 production and neuroinflammation [38]
AD	lncMEG3↓	PI3K/Akt	Improves cognitive impairment, alleviates neuronal damage [39]
AD	lncUCA1↑	miR-1/Hes1	Regulates neural stem cell differentiation [40]
AD	circNF1-419↑	Dynamin-1, AP2B1	Enhances astrocytes autophagy to ameliorate senile dementia [41]
PD	miR-155↑		Promotes microgliosis and proinflammatory response [48]
PD	miR-7116-5p↓	TNF-α	Suppresses TNF-α production and inflammatory responses [49]
PD	miR-335↓	LRRK2	Alleviates inflammatory responses [50]
PD	miR-124↓	MEKK3	Attenuates microglia activation and neuroinflammation [51]
PD	miR-124↓	p38, p62	Attenuates microglia activation and neuroinflammation [52]
PD	miR-3473b↑	TREM2	Promotes the activation of microglia and inhibits autophagy [53]
PD	miR-19a-3p↑	PTEN/AKT/mTOR	Suppress autophagy in recipient microglia [54]
PD	miR-7↓	NLRP3	Modulates NLRP3 inflammasome-mediated inflammation [55]
PD	lncSNHG1↑	miR-7/NLRP3	Promotes microglia activation and NLRP3 inflammasome [56]
PD	lncGAS5↑	miR-223-3p/NLRP3	Promotes microglial inflammatory response [57]
PD	lncMALAT1↑	EZH2/NRF2	Promotes inflammasome activation and ROS production [58]
PD	lncHOXA11-AS↑	miR-124-3p/FSTL1	Enhances microglia activation and neurological damages [59]
PD	lincRNA-p21↑	miR-181/PKC-δ	Facilitates sustained microglial activation [60]
ALS	miR-155↑		Promotes dysfunctional microglia and deteriorate disease [65]
ALS	miR-125b↑	A20	Promotes microglia activation and motor neuron death [66]
ALS	miR-146a↓	IRAK1, TRAF6	Reverts astrocytes aberrancies [67]
ALS	miR-124↓	Sox2, Sox9	Modulates astrocytic differentiation of neural stem cells [68]
ALS	miR-494-3p↓	SEMA3A	Increases MN survival [70]
ALS	miR-218↑	EAAT2	Promotes astrogliosis [71]
MS	miR-155↑	CD47	Promote neuroinflammation [75]
MS	miR-155↑	SOCS1	Promotes inflammatory responses in microglia and astrocytes [77]
MS	miR-155↑	Annexin-2, Claudin-1, DOCK-1, Syntenin-1	Negatively regulates BBB function [78]
MS	miR-146a↑	IRAK1	Promotes OPC differentiation and enhances remyelination [79]
MS	miR-146a↑	Syt1, Nlg1	Decreases density of dendritic spines and excitatory synapses [80]
MS	miR-223↑	ATG16L1	Suppresses autophagy and promotes CNS inflammation [81]
MS	miR-223↑	RASA1	Promotes regenerative myeloid cell phenotype and function [82]
MS	miR-124↓	C/EBP-α-PU.1	Promotes microglia quiescence and suppresses EAE [83]
MS	miR-30a↑	Ppargc1b	Promotes microglial inflammatory response and aggravates EAE [84]
MS	miR-23b↓	TAB2, TAB3, IKK-α	Suppresses IL-17-associated autoimmune inflammation [86]
MS	miR-873↑	A20/NF-κB	Contributes to the inflammatory response and EAE pathogenesis [87]
MS	miR-409-3/1896↑	SOCS3/STAT3	Promotes inflammatory cytokine production and EAE pathogenesis [88]
MS	lncGm13568↑	CBP/P300	Contributes to the pathogenesis of EAE [89]
MS	lncGAS5↓	PRC2	Inhibits microglial M2 polarization and exacerbates demyelination [90]
MS	lncHOTAIR↓	miR-136-5p/AKT2	Promotes M1 microglial polarization and suppresses remyelination [91]

### Parkinson’s disease

Parkinson’s disease (PD) is the second most common neurodegenerative disease after AD, and its symptoms include resting tremors, bradykinesia, rigidity, and postural instability. The main pathological features of PD are the loss of dopaminergic neurons and intracellular inclusions containing a-synuclein, called Lewy bodies and Lewy neurites [26]. PD was originally defined as a disease characterized by the loss of dopaminergic neurons; however, an increasing number of studies have shown that PD has an inflammatory component [42–44]. Microglia can phagocytose extracellular a-synuclein, and aggregated a-synuclein promotes microglial activation and induces inflammatory responses, which play essential roles in the pathogenesis of PD [45]. In addition, inflammatory mediators secreted by astrocytes and microglia, such as proinflammatory cytokines, reactive oxygen species (ROS), and nitric oxide (NO), regulate the progression of neuronal cell death in PD [46].

Microglial activation leads to exacerbated inflammatory responses in PD, and miRNAs are important regulators of this process [47]. miR-155 was significantly upregulated in a PD mouse model. Its knockout attenuates α-synuclein-induced microgliosis and neurodegeneration, whereas treatment of miR-155 knockout microglia with miR-155 mimics restores the proinflammatory response [48]. In another PD mouse model induced by 1-methyl-4-phenyl-1,2,3,6-tetrahydropyridine (MPTP), miR-7116-5p was downregulated in microglia. Overexpression of miR-7116-5p suppressed microglial activation by targeting tumor necrosis factor α (TNF-α) [49]. Similarly, the expression of miR-335 decreased in a PD model, and miR-335 attenuated proinflammatory responses in microglia by inhibiting leucine-rich repeat kinase 2 (LRRK2) [50]. miR-124 attenuates microglial activation in MPTP-induced PD models. miR-124 inhibits the NF-κB signaling pathway by depleting MEKK3 [51], targets p38 and p62, and promotes autophagy during the inflammatory pathogenesis of PD [52]. Autophagy is closely associated with microglial activation, which attenuates microglial activation and suppresses proinflammatory responses. TREM2 inhibits autophagy via mTOR signaling. However, TREM2 was identified as a target of miR-3473b in an MPTP-induced PD model. miR-3473b antagomir treatment inhibited microglial activation and promoted autophagy [53]. Furthermore, the phosphatase and tensin homolog (PTEN), an inducer of autophagy, is targeted by miR-19a-3p in microglia. Exosome overloading with miR-19a-3p suppresses autophagy in recipient microglia by targeting the PTEN/AKT/mTOR pathway [54].

The NOD-like receptor protein 3 (NLRP3) inflammasome is highly expressed in microglia and has been implicated in PD pathogenesis. Importantly, miR-7 targets NLRP3 expression and inhibits NLRP3 inflammasome activation. The injection of miR-7 mimics into PD model mice attenuated microglial activation and dopaminergic neuron degeneration [55]. Furthermore, lncRNA small nucleolar RNA host gene 1 (lncSNHG1) functions as a competing endogenous RNA (ceRNA) for miR-7 to modulate NLRP3 expression, which promotes microglial activation and the NLRP3 inflammasome [56]. In addition, lncRNA growth arrest-specific 5 (lncGAS5) modulates NLRP3 expression in both in vivo and in vitro models of PD and upregulates NLRP3 via competing miR-223-3p [57]. In addition to functioning as ceRNAs, lncRNAs regulate inflammasomes by binding to proteins. In an MPTP-induced PD model, MALAT1 recruits the enhancer of zeste homolog 2 (EZH2) to the nuclear factor-like-2 (NRF2) promoter and inhibits NRF2 expression, thereby facilitating inflammasome activation in PD microglia and mouse models [58]. In addition, lncRNAs regulate neuroinflammation by modulating NF-κB signaling in PD. Follistatin-like 1 (FSTL1) is an activator of NF-κB signaling pathways, and reduction of lncHOXA11-AS inhibits FSTL1 expression by upregulating miR-124-3p, thereby attenuating microglial activation and neurological damage in PD models [59]. PKC-δ, which can regulate NF-κB activation, is upregulated by lincRNA-p21 by sponging miR-181. Interestingly, p53, lincRNA-p21, miR-181, and PKC-delta form a double-negative feedback loop that promotes persistent microglial activation in PD [60] (Fig. 1, Table 1).

### Amyotrophic lateral sclerosis

Amyotrophic lateral sclerosis (ALS) is a fatal adult-onset neurodegenerative disease that affects motor neurons (MN) in the brainstem, spinal cord, and motor cortex [61]. The clinical features include muscle stiffness and twitching, limb weakness, cognitive impairment, and hyperreflexia. Once considered a motor neuron disease, ALS is now recognized as a multisystem, multicellular disorder [62]. Neuroinflammation is readily observed in imaging studies of human patients with ALS and rodent ALS models and is characterized by microgliosis and astrocytosis. Activated microglia and astrocytes can further damage the MN and contribute to neuronal death by secreting proinflammatory cytokines and apoptosis-inducing molecules [26].

Mutations in superoxide dismutase 1 (SOD1) are involved in the pathogenesis of ALS. The SOD1 mouse, which expresses human SOD1 protein with ALS mutations, can recapitulate the disease and is the most widely used model for ALS [63]. A recent study showed alterations in neuroprotective and neuroinflammatory effects in the spinal cord of SOD1 mice. In the symptomatic stage, glial function is significantly depressed, cell-to-cell communication is reduced, and miR-155 is upregulated. In contrast, astrocytosis, microgliosis, and neuroinflammation are evident during the symptomatic stages. Moreover, several inflammation-related miRNAs, including miR-155, miR-146a, miR-125b, miR-124, and miR-21, were significantly upregulated, suggesting that these miRNAs may play essential roles in modulating neuroinflammation in ALS [64]. Indeed, miRNAs are widely involved in the regulation of microglial- and astrocyte-mediated inflammatory responses in ALS. In a SOD1 mouse model, miR-155 knockout reversed the abnormal molecular signature and phagocytic function of microglia. In addition, treatment of SOD1 mice with anti-miR-155 delayed disease onset and prolonged survival [65]. The anti-inflammatory protein A20 is induced in microglia upon inflammatory BzATP stimulation, and A20 is regulated by miR-125b. Inhibition of miR-125b suppresses NF-κB activation and the release of toxic factors, thereby protecting MN from death induced by activated microglia [66]. The expression of miR-146a is decreased in astrocytes isolated from the cortex of symptomatic SOD1 (mSOD) mice, whereas its target genes IRAK1 and TRAF6 are upregulated. Overexpression of pre-miR-146a in mSOD astrocytes attenuates the aberrant phenotype of astrocytes, including the restoration of GFAP, S100B, vimentin, Cx43, and HMGB1. In contrast, the transfection of anti-miR-146a into wild-type astrocytes reproduces the aberrant phenotype of mSOD1 astrocytes [67]. miRNAs also regulate the differentiation of neurons and astrocytes. miR-124 is downregulated in the spinal cord and brainstem of SOD1 mice and is associated with the astrocytic differentiation of NSCs by targeting Sox2 and Sox9 [68].

Glial cells normally support neurons; however, in ALS, the homeostatic role of glial cells is often lost and replaced by deleterious effects on neurons. Glial cells may respond to the dying neurons and exacerbate their degeneration [69]. Therefore, neuron–glial crosstalk is of great importance in ALS, and miRNAs are critical regulators of this process. A recent study showed that astrocytes from patients with ALS are toxic to MN and that this toxicity is mainly mediated by astrocyte-derived extracellular vesicles (ADEV). Moreover, miR-494-3p was significantly downregulated in ADEV and regulated its target gene SEMA3A in MN. SEMA3A contributes to reduced neurite growth and MN death in ALS. Therefore, reduced miR-494-3p expression in ADEV leads to MN loss and ALS pathogenesis [70]. In addition, MN-derived miRNAs directly modulate glial cell phenotypes. MN-specific miR-218 is highly enriched in MN and is released extracellularly in ALS rat models. Astrocytes that do not express miR-218 take up extracellular miR-218 released from dying MN, and miR-218 downregulates the glutamate transporter EAAT2 in astrocytes, thus contributing to astrogliosis and progressive neuronal damage [71] (Fig. 1, Table 1).

### Multiple sclerosis

Multiple sclerosis (MS) is a chronic neurodegenerative disease of the central nervous system (CNS) that occurs primarily in young adults [72]. It is mainly considered an autoimmune disease characterized by inflammation and demyelination of neurons. The progressive pathological processes of MS include BBB breakdown, multifocal inflammation, reactive microgliosis, astrocytosis, demyelination, and axonal degeneration [73]. Increasing evidence suggests that MS progression and symptoms are closely correlated with the maintenance of persistent, low-grade inflammation driven by microglia and astrocytes [74].miRNAs are important modulators of microglial- and astrocyte-mediated inflammatory responses in MS. miRNA profiling shows that 20 miRNAs, including miR-155, miR-146a, miR-223, miR-142, miR-34a, and miR-326, are highly upregulated in active MS lesions, among which miR-155 regulates neuroinflammation in MS [75]. miR-155 promotes proinflammatory responses in microglia and astrocytes by targeting SOCS1 [76, 77]. In addition to its direct proinflammatory effect on glial cells, miR-155 can exacerbate neuroinflammation in MS by targeting focal adhesions and tight junctions, resulting in increased BBB permeability and peripheral leukocyte infiltration into the CNS [78]. In contrast, miR-146a has a heterogeneous role in MS. In an experimental autoimmune encephalomyelitis (EAE) mouse model, administration of miR-146a mimics facilitated M2 microglial polarization and promoted oligodendrocyte progenitor cell (OPC) differentiation and remyelination, suggesting the therapeutic functions of miR-146a in MS [79]. Furthermore, inflammatory microglial transfer of miR-146a to neurons via extracellular vesicles (EVs) leads to a significant decrease in the density of dendritic spines and excitatory synapses, and sustained exposure to miR-146a-enriched EVs results in pathological synapse loss and synaptic dysfunction [80]. The role of miR-223 in inflammatory responses is also controversial in MS. In an EAE model, miR-223 knockout ameliorated pathogenic CNS inflammation, demyelination, and clinical symptoms of EAE [81]. Mechanistically, miR-223 inhibits autophagy by targeting ATG16L1; therefore, miR-223 deficiency promotes microglial autophagy and increases the number of resting microglia. Moreover, miR-223 deficiency leads to delayed onset of EAE, but the disease severity differed [82]. miR-223 deficiency has little effect on the proinflammatory phenotype of microglia and macrophages; however, miR-223 is essential for M2 polarization and phagocytosis of microglia and macrophages, and miR-223 knockout impairs CNS remyelination and myelin debris clearance. miR-124 and miR-30a also regulate the inflammatory response of microglia to EAE. In vivo administration of miR-124 reduces EAE progression by promoting microglial quiescence and deactivating macrophages by targeting the C/EBP-α-PU.1 pathway [83]. Conversely, miR-30a contributes to the microglial inflammatory response, and transplantation of miR-30a-modified microglia exacerbates EAE progression [84].

The inflammatory cytokine interleukin-17 (IL-17) is a key regulator in autoimmune diseases. Increasing evidence indicates that miRNAs are broadly involved in the IL-17-mediated proinflammatory response in MS [85]. miR-23b is one of the prominent miRNAs involved in IL-17-associated autoimmune inflammation in MS. IL-17 decreases the expression of miR-23b in astrocytes, thereby inhibiting the expression of inflammatory cytokines by targeting TAB2, TAB3, and IKK-α. Therefore, IL-17 exacerbates MS progression by attenuating miR-23b expression and increasing proinflammatory cytokine expression [86]. In an EAE model, IL-17 increases miR-873 level in astrocytes and mouse brain tissue, which enhances the expression of inflammatory cytokines and exacerbates MS progression via regulation of the A20/NF-κB pathway [87]. Similarly, miR-409-3p and miR-1896 induced by IL-17 stimulation coordinately promote proinflammatory responses in reactive astrocytes by modulating the SOCS3/STAT3 pathway [88].

Emerging evidence suggests that IL-9 plays a regulatory role in autoimmune responses in MS. A recent study characterized an IL-9-triggered lncRNA, Gm13568, in astrocytes, which promotes proinflammatory responses in active astrocytes and contributes to EAE pathogenesis. Gm13568 interacts with CBP/P300 and stimulates Notch1 pathway activation, thereby increasing the production of inflammatory cytokines [89]. lncRNAs also modulate microglial polarization in MS. Microarray screening shows that 120 lncRNAs are differentially expressed in M2-polarized microglia versus resting microglia, among which the lncGAS5 is significantly downregulated. lncGAS5 inhibits TRF4 transcription by recruiting PRC2, thereby attenuating M2 microglial polarization. Knockdown of lncGAS5 in transplanted microglia ameliorates EAE progression and facilitates remyelination [90]. Sulfasalazine (SF) is an anti-inflammatory drug that improves outcomes in patients with MS. A recent study has shown that SF suppresses M1 microglial polarization and promotes remyelination. Mechanistically, SF blocked AKT2-NF-κB signaling through the effects of lncHOTAIR and miR-136-5p in microglia [91] (Fig. 1, Table 1).

### Ischemic stroke

Ischemic stroke (IS) is a leading cause of death and disability worldwide, resulting from the occlusion of a cerebral artery that interrupts cerebral blood flow and causes rapid loss of brain function [92]. Reperfusion therapy is an effective therapeutic approach for reducing primary injury; however, it can also induce cerebral ischemia–reperfusion (I/R) injury, which causes secondary neuronal damage and death [93]. Post-IS inflammation mediated by microglia and astrocytes plays a dual role in brain tissue damage and repair [94]. Microglia are rapidly activated after IS and produce proinflammatory cytokines that cause tissue injury. In contrast, M2-phenotype microglia release anti-inflammatory cytokines that contribute to functional recovery after IS. Reactive astrogliosis may exacerbate ischemic lesions and hinder axonal regeneration. However, it also contributes to neuroprotection and neurological recovery [95].miRNAs are involved in the regulation of various pathogenic mechanisms underlying tissue injury after stroke, including inflammatory responses, excitotoxicity, oxidative stress, mitochondrial dysfunction, and BBB dysfunction [96]. Importantly, miRNAs are involved in glia-mediated inflammation by modulating the activation, polarization, proliferation, and apoptosis of microglia and astrocytes. Several miRNAs have been shown to ameliorate glia-mediated inflammatory responses [97]. miR-146a is significantly upregulated in microglia after oxygen–glucose deprivation (OGD) [98], and miR-146a-loaded exosomes can inhibit microglial activation and neuroinflammation via the IRAK1/TRAF6 pathway [93]. Similarly, miR-1906 and miR-669c can ameliorate post-IS neuroinflammation by modulating the TLR signaling pathway; miR-1906 directly targets TLR4, whereas miR-669c inhibits the canonical adaptor protein MyD88, thereby blocking TLR4 signaling and reducing inflammatory responses [99, 100]. miR-124 contributes to neuroprotection and functional recovery and may serve as a promising candidate for treating IS [101]. Recent studies further support the beneficial role of miR-124 in IS; miR-124 transported by M2 microglia-derived exosomes can suppress neuronal apoptosis and inhibit glial scar formation, thereby improving the outcome of IS [102, 103]. Similarly, miR-424 attenuates brain injury and promote functional recovery after IS, and its effects are mediated by attenuating microglial activation and astrogliosis [104, 105]. In addition, overexpression of let-7c-5p inhibits microglial activation and attenuates brain damage after IS by targeting caspase 3 [106]. Microglial phagocytosis of stressed but viable neurons is considered detrimental to the brain, whereas EV-derived miR-98 can prevent microglial phagocytosis of salvageable neurons by targeting PAFR, thereby reducing neuronal death during IS [107]. Moreover, miR-98 protects the BBB from proinflammatory monocyte infiltration, thereby preventing further microglial activation and improving neurological outcomes after IS [108].

However, miRNAs can also aggravate IS-induced brain injury. The miR-15a/16-1 cluster has opposite regulatory roles to miR-98 in BBB permeability; knockout of the miR-15a/16-1 cluster attenuates peripheral immune cell infiltration and inhibits M1 microglia [109]. In a mouse model of middle cerebral artery occlusion (MCAO), miR-3473b levels increased in the cortex and striatum. Upregulation of miR-3473b exacerbates the pathogenesis of IS by promoting microglia-mediated neuroinflammatory injury, and the associated mechanism involves the regulation of SOCS3 [110]. In another MCAO model, miR-181c aggravated brain ischemia–reperfusion injury by promoting microglial and neuronal apoptosis by regulating apoptosis-related genes, including BCL-2 and BAX [111]. miR-148b and miR-365 modulate neurogenesis in IS; miR-148b inhibits the proliferation and differentiation of NSC into neurons and astrocytes by regulating Wnt/β-catenin signaling, thereby attenuating the recovery of neurological function after stroke [112]. miR-365 suppresses the conversion of astrocytes into mature neurons and subsequently exacerbates ischemic injury by targeting PAX6 [113].lncRNAs and circRNAs have recently emerged as critical modulators of IS. The dysregulation of lncRNAs is involved in regulating microglial inflammation by its effects on microglial activation and polarization [114, 115]. In a mouse model of MCAO, overexpression of lnc1810034E14Rik inhibited microglial activation by reducing p65 phosphorylation, thereby alleviating brain damage [116]. lncRNAs NEAT1, H19, TUG1, and GAS5 can shift microglial polarization in IS, and lncNEAT1 attenuates M1 microglial polarization to suppress OGD/R-induced injury [117]. In contrast, lncH19 promotes M1 microglial polarization and contributes to neuroinflammation [118]. Similar to H19, lncTUG1 can facilitate microglial polarization toward the M1 phenotype, and this process is mediated by miR-145a-5p [119]. lncGAS5 increases M1 microglial polarization and decreases M2 microglial polarization by upregulating Notch1 expression via miR-146a [120]. In addition, lncRNAs are involved in glial cell apoptosis. Downregulation of MALAT1 inhibits astrocyte apoptosis and protects against cerebral I/R injury; the associated mechanism is related to miR-145 and its target AQP4 [121]. In contrast, lncNespas knockdown exacerbates the I/R-induced microglial apoptosis and inflammatory responses. lncNespas can block the interaction between TRIM8 and TAK1 and attenuate the K63-linked polyubiquitination of TAK1, resulting in the inactivation of TAK1 and NF-κB signaling [122]. Moreover, microglial activation-induced lncRNA U90926 promotes neutrophil infiltration via a mechanism involving the regulation of CXCL2. U90926 interacts with MDH2 and prevents the binding of MDH2 to the CXCL2 3ʹ UTR, thereby inhibiting MDH2-mediated decay of CXCL2 mRNA [123]. lncRNAs also serve as therapeutic targets for IS by regulating NSC differentiation. Furthermore, inhibiting lncPnky in NSCs promotes their differentiation into neurons and astrocytes, leading to improved functional recovery after IS [124]. In addition, circRNAs are involved in IS-associated autophagy. In a mouse model of MCAO, circHECTD1 was highly expressed and significantly upregulated in the ischemic brain tissue. circHECTD1 promotes autophagy in astrocytes by targeting the miR-142/TIPARP axis [125]. circSHOC2 is highly expressed in astrocyte-derived exosomes. It functions as an endogenous miR-7670-3p sponge to regulate SIRT1 expression, resulting in reduced neuronal autophagy and ameliorating neuronal damage after IS [126] (Fig. 1, Table 2).

**Table 2:** ncRNAs in the regulation of astrocyte- and microglia-mediated neuroinflammation in acute neurodegenerative disease

	ncRNAs	Target	Function
IS	miR-146a↑	IRAK1/TRAF6	Reduces microglial-mediated neuroinflammation [93]
IS	miR-1906↑	TLR4	Inhibits poststroke inflammation and ameliorates brain injury [99]
IS	miR-669c↑	MyD88	Modulates microglial/macrophage toward anti-inflammatory phenotype [100]
IS	miR-124↑	USP14	Induces neuroprotection and functional improvement [101, 102]
IS	miR-424↓	NFIA	Inhibits microglia activation and astrogliosis [104, 105]
IS	let-7c-5p↓	Caspase 3	Suppresses microglia activation [106]
IS	miR-98↑	PAFR	Prevents salvageable neurons from microglial phagocytosis [107]
IS	miR-98↑		Protects BBB and improves neurological outcomes [108]
IS	miR-15a/16-1↑	Claudin-5	Exacerbate BBB dysfunction [109]
IS	miR-3473b↑	SOCS3	Enhances post-stroke neuroinflammation injury [110]
IS	miR-181c↓	BCL-2, BAX	Promotes apoptosis of microglia and neurons [111]
IS	miR-148b↑	Wnt/β-catenin	Attenuates proliferation and differentiation of NSC [112]
IS	miR-365↑	PAX6	Inhibits astrocyte-to-neuron conversion [113]
IS	lnc1810034E14Rik↑	p65	Reduces microglia activation and alleviates brain damage [116]
IS	lncNEAT1↑	AKT/STAT3	Inhibits M1 microglial polarization [117]
IS	lncH19↑	TNF-α, CD11b	Promotes M1 microglial polarization [118]
IS	lncTUG1↑	miR-145a-5p	Promotes M1 microglial polarization [119]
IS	lncGAS5↑	miR-146a/Notch1	Suppresses microglial M2 polarization and promotes M1 polarization [120]
IS	lncMALAT1↑	miR-145/AQP4	Increases astrocytes apoptosis [121]
IS	lncNespas↑	TAK1/TRIM8	Inhibits microglia apoptosis and neuroinflammation [122]
IS	lncU90926↑	MDH2/CXCL2	Facilitates neutrophil infiltration [123]
IS	lncPnky↓	PTBP1	Promoted the differentiation of NSCs into neurons and astrocytes [124]
IS	circ-HECTD1↑	miR-142/TIPARP	Promotes astrocyte autophagy [125]
IS	circ-SHOC2↑	miR-7670-3p/SIRT1	Inhibits neuronal autophagy and ameliorates ischemic brain injury [126]
TBI	miR-142↑		Contributes to astrocyte activation and brain inflammation [129]
TBI	miR-155↑		Contributes to progressive neuroinflammatory responses [130, 131]
TBI	miR-200b↓	cJun/MAPK	Inhibits microglial inflammatory responses [132]
TBI	miR-17-92↑	GP130, CNTFR, JAK2, STAT3	Promotes neuronal differentiation of grafted NSCs [133]
TBI	miR-9-5p↑	Thbs-2	Promotes astrocyte proliferation and synaptic remodeling [134]
TBI	miR-124-3p↑	Rela/ApoE	Alleviates neurodegeneration and improves cognitive outcome [136]
TBI	miR-124-3p↑	PDE4B/mTOR	Inhibits neuronal inflammation and contributes to neurite outgrowth [137]
TBI	miR-21-5p↑		Promotes polarization of M1 microglia [138]
TBI	miR-873a-5p↑	NF-κB	Attenuates microglia-mediated neuroinflammation [139]
TBI	lncMeg3↑	miR-7a-5p/Nlrp3	Promotes microglial activation and inflammation [141]
TBI	lncKCNQ1OT1↑	miR-873-5p/TRAF6	Facilitates M1 microglia polarization [142]
TBI	lncHOTAIR↑	MYD88	Promotes microglia activation and inflammatory factor release [143]
TBI	lncGm4419↑	miR-466l/TNF-α	Promotes trauma-induced astrocyte apoptosis [144]
TBI	lncMalat1↓	IL-6, AQP4	Reduces astrocyte swelling and ameliorates brain edema [145]
TBI	lncNKILA↑	miR-195/NLRX1	Alleviates neuronal injury [146]
SCI	miR-17-5p↑	JAK/STAT3	Promotes astrocyte proliferation [150]
SCI	miR-145↓	GFAP, c-myc	Inhibits astrogliosis [151]
SCI	miR-21↑	PTEN	Regulates astrocytic function and promotes the functional recovery [152]
SCI	miR-124↑	MYH9	Inhibits microglia activation and phagocytic activity [154, 155]
SCI	miR-216a-5p↑	TLR4	Shifts microglia from the M1 to M2 phenotype [157]
SCI	miR-340-5p↓	P38	Ameliorates SCI-induced neuroinflammation and apoptosis [158]
SCI	lncZeb2os↑	Zeb	Promotes reactive astrogliosis [159, 160]
SCI	lncGm37494↑	miR-130b-3p/PPARγ	Shifts microglial M1/M2 polarization [161]
SCI	lncMALAT1↑	miR-199b/IKKβ	Contributes to inflammatory response of microglia [162]
SCI	lncXIST↑	miR-27a/Smurf1	Promotes the apoptosis and inflammatory injury of microglia [163]
SCI	lncFtx↓	miR-382-5p/Nrg1	Reduces the inflammation response of microglia [164]
SCI	lncLEF1-AS1↑	miR-222-5p/RAMP3	Promotes apoptosis and inflammatory injury of microglia [165]
SCI	lncCCAT1↓	miR-218/NFAT5	Alleviating apoptosis and inflammation damage of astrocytes [166]
SCI	lncF630028O10Rik↑	miR-1231-5p/Col1a1	Enhances microglial pyroptosis [167]
SCI	lncSNHG5↑	KLF4	Enhances astrocytes and microglia viability [168]
SCI	circRNA_01477↓	miR-423-5p	Promotes astrocyte proliferation and migration [169]

### Traumatic brain injury

Traumatic brain injury (TBI) is a leading cause of mortality in developed countries. The pathology of TBI is complex and can be divided into two main stages: primary and secondary injuries. Primary injury occurs during lesion formation, resulting in contusion and hemorrhage. Secondary injury is caused by the complicated processes of initial impact and is characterized by various neuropathological processes, including ischemia, oxidative stress, excitotoxicity, apoptosis, necrosis, and neuroinflammation [127]. Among these, neuroinflammation is an important pathological process, and microglia and astrocytes are considered critical players in initiating inflammatory responses and determining the extent of damage during TBI [128].

In a TBI rat model, the expression of miR-155 and miR-142 was highly upregulated in the perilesional cortex; miR-155 was predominantly expressed by activated astrocytes, whereas miR-142 expression was associated with microglia, macrophages, and lymphocytes. miR-155 and miR-142 contribute to astrocyte activation and brain inflammation after TBI [129]. In particular, miR-155 plays a critical role in progressive neuroinflammatory responses in TBI. Microglia-derived microparticles loaded with proinflammatory mediators, including miR-155, promote microglial activation and persistent neuroinflammatory responses [130]. Moreover, miR-155 inhibition suppresses post-traumatic neuroinflammatory responses and improves neurological recovery after TBI in mice [131]. miR-200b modulates microglial inflammatory processes in TBI. The expression of miR-200b is decreased in activated microglia, and the downregulation of miR-200b leads to increased inflammatory responses via cJun/MAPK signaling [132]. In addition, miRNAs modulate astrocyte differentiation and proliferation in TBI. Activated astrocytes can regulate NSC differentiation by secreting various cytokines, such as leukemia inhibitory factor (LIF) and ciliary neurotrophic factor (CNTF), which promote the premature generation of astrocytes via the activation of JAK and STAT signaling pathway. However, several proteins involved in JAK2/STAT3 signaling are direct targets of the miR-17-92 cluster. Therefore, the miR-17-92 cluster suppresses astrocytogenesis and increases neurogenesis, improving motor coordination as observed in brain-injured mice [133]. Furthermore, miR-9-5p is significantly upregulated in brain tissue after TBI. Inhibition of miR-9-5p upregulates the expression of its target gene, Thbs-2, in astrocytes, thereby promoting the proliferation of astrocytes and the release of astrocyte-derived neurotrophic factors, leading to the recovery of neurological function [134].

Exosomes are associated with the cell-to-cell crosstalk involved in immune regulation in TBI, and several exosome-carried miRNAs have been shown to modulate TBI neuroinflammation [135]. In TBI, miRNA-loaded exosomes are involved in communication between glia and neurons. In a model of repetitive mild TBI, the expression of miR-124-3p is significantly upregulated in microglial exosomes. Treatments with miR-124-3p-loaded exosomes promote anti-inflammatory M2 polarization in microglia and alleviate neurodegeneration by targeting the Rela/ApoE signaling pathway [136]. A similar study involving TBI shows that Exo-miR-124-3p contributes to M2 microglial polarization and attenuates neuronal inflammation via transfer into neurons; these effects are mediated by PDE4B/mTOR signaling [137]. Neuron-derived exosomes regulate glial inflammatory responses in TBI. For example, neuron-derived exosomes loaded with miR-21-5p are phagocytosed by microglia and promote M1 microglial polarization [138]. In addition, miRNA-loaded exosomes are involved in the cellular communication between astrocytes and microglia. Moreover, activated astrocyte-derived exosomes are enriched with miR-873a-5p, which can suppress microglial inflammatory responses by promoting M2 microglial polarization after TBI [139].

Several lncRNAs are implicated in the onset and progression of TBI. lncRNAs modulate microglial inflammation in TBI [140]. For example, lncMEG3 facilitates Nlrp3-mediated microglial activation and inflammatory responses by targeting miR-7a-5p [141]. The lncRNA KCNQ1OT1 serves as an endogenous miRNA sponge for miR-873-5p, thereby promoting TRAF6 expression and ultimately contributing to M1 microglial polarization [142]. HOTAIR is highly expressed in activated microglia, where it binds to the MYD88 protein and increases its stability by inhibiting Nrdp1-mediated ubiquitination of MYD88. Downregulation of HOTAIR suppresses microglial activation and the release of inflammatory factors [143]. In addition, lncRNAs are involved in regulating astrocytic inflammation in TBI. lncGm4419 level increases significantly in astrocytes after TBI and can enhance TNF-α expression by competitively binding to miR-466l to promote astrocyte apoptosis [144]. Cerebral edema, an important neurological complication of TBI, is characterized by early astrocyte swelling. MALAT1 reduces astrocyte swelling and improve brain edema; its effects are mediated by reducing AQP4 and IL-6 expression [145]. In addition, astrocyte-derived EVs carrying the lncRNA NKILA contribute to the recovery of injured neurons after TBI. Mechanistically, NKILA promoted NLRX1 expression by competitively binding to miR-195 [146] (Fig. 1, Table 2).

### Spinal cord injury

Spinal cord injury (SCI) is a major cause of morbidity and mortality worldwide, resulting in long-term cognitive or motor dysfunction [147]. Similar to TBI, mechanical insults induce primary damage to the spinal cord, and a series of pathological cascades cause secondary damage, leading to neuronal and glial cell death, inflammation, and ischemia [148].

Astrogliosis is characterized by astrocyte proliferation and hypertrophy, resulting in the formation of glial scars that act as physical barriers to axonal regeneration. However, in the early stages of SCI, astrogliosis is essential for preventing advanced injury [149]. Various miRNAs have been shown to modulate the astrocyte phenotype and function in SCI. The RNase III ribonuclease Dicer1 is required for the formation of mature miRNAs, and deletion of Dicer1 prevents astrocyte proliferation after SCI. However, treatment with the miR-17-5p mimic corrects the defective proliferation of Dicer1 knockout astrocytes. Furthermore, the suppression of miR-17-5p can prevent lipopolysaccharide-induced astrocyte proliferation [150]. The levels of astrocyte-enriched miR-145 decrease after SCI, and the downregulation of miR-145 in astrocytes promotes astrocyte growth, motility, and hypertrophy by enhancing GFAP and c-myc expression, which is beneficial for spinal cord tissue repair [151]. In addition, miR-21 expression is significantly upregulated in response to SCI. Overexpression of miR-21 in astrocytes contributes to astrocyte secretion and proliferation and promotes recovery after SCI; these effects are mediated by PTEN/ PI3K/Akt/mTOR signaling [152].

Activated microglia promote secondary tissue damage in SCI. Similarly, miRNAs have been implicated in the modulation of microglial activation and polarization in SCI [153]. miR-124 is a well-studied miRNA that regulates microglial response after SCI. In a rat SCI model, the delivery of miR-124 to rat microglia inhibited microglial activation and subsequently attenuated neuroinflammation, suggesting that miR-124 is a therapeutic target for suppressing inflammation in SCI [154]. In addition, neuron-derived exosomes loaded with miR-124 facilitated functional behavioral recovery in SCI by inhibiting the activation of microglia and astrocytes [155]. In addition, miR-124 regulates the phagocytic response of microglia. Moreover, docosahexaenoic acid (DHA) exerts neuroprotective effects in SCI, and these effects are mediated by a miR-124-dependent reduction in microglial phagocytic activity [156]. Similarly, exosomes secreted by mesenchymal stem cells (MSCs) facilitate functional behavioral recovery by modulating microglial activation. Exosome-carried miR-216a-5p shifts microglia from the M1 to the M2 phenotype by targeting the TLR4/NF-κB/PI3K/AKT signaling pathways [157]. In addition, miR-340-5p ameliorates neuroinflammation and promotes functional recovery after SCI. Mechanistically, miR-340-5p targets P38 and inhibits the MAPK pathway to ameliorate the SCI-induced inflammatory responses [158].

Interestingly, both a contusive SCI mouse model and RNA-seq of the SCI epicenter in the acute and chronic stages showed that the protein-coding gene Zeb2 and its antisense lncRNA Zeb2os were upregulated and colocalized in astrocytes. Knockdown of Zeb2 and lncZeb2os attenuated reactive astrogliosis, demonstrating similar roles of Zeb2 and lncZeb2os in the astrocytic response after SCI. lncZeb2os can positively regulate Zeb2 expression, and these effects may be mediated by RNA–RNA, RNA–DNA, or RNA–protein interactions [159, 160]. Importantly, several lncRNAs (including lncGm37494, lncMALAT1, lncXIST, lncFtx, lncLEF1-AS1, lncCCAT1, and lncF630028O10Rik) have been confirmed as endogenous miRNA sponges that regulate the inflammatory responses of microglia and astrocytes after SCI [161–167]. For example, TLR4-induced lncF630028O10Rik promotes microglial pyroptosis after SCI by targeting the miR-1231-5p/Col1a1 axis, thus providing a therapeutic target for attenuating neuroinflammation in SCI [167]. In addition, lncRNAs interact with proteins to regulate SCI. KLF4 is a conserved transcription factor involved in microglial and astrocytic activation in SCI. lncSNHG5 can directly bind to KLF4 and increase its expression, thereby facilitating the viability of microglia and astrocytes [168]. circRNAs have also been implicated in the regulation of SCI progression. A recent circRNA profile in an SCI rat model showed that 360 circRNAs were differentially expressed in the spinal cord tissues, among which circRNA_01477 expression was significantly decreased. Downregulation of circRNA_01477 suppresses astrocyte proliferation and migration [169] (Fig. 1, Table 2).

### Viral encephalitis

Although the CNS is not a common viral target, many viruses can infect the brain and induce neuroinflammation, which is characterized by severe neuronal injury and microgliosis/astrogliosis. Microglia and astrocytes actively respond to both RNA and DNA viruses by releasing various inflammatory mediators to activate the immune system and combat infection [170–172].

Japanese encephalitis virus (JEV) is a mosquito-borne ssRNA virus that causes acute inflammatory diseases of the CNS called Japanese encephalitis (JE). JE is the most common viral encephalitis in the Asia–Pacific region, killing one-third of patients and leaving nearly half of the survivors with permanent neuropsychiatric sequelae [173]. JEV directly infects microglia and astrocytes and induces glial activation. Accumulating evidence has implicated miRNAs as essential regulators of microglia-mediated neuroinflammation in JEV infections. In response to JEV infection, miR-146a was significantly upregulated in microglia and functioned as a negative feedback regulator in JEV-induced inflammatory responses [174]. miR-146a also facilitated JEV replication by inhibiting NF-κB activity and antiviral JAK–STAT signaling [175]. miR-155 is also upregulated after JEV infection; however, its role remains controversial. One study showed that increased miR-155 expression further exacerbates JEV-induced neuroinflammation by targeting SHIP1 [176]. Another study showed that miR-155 inhibited microglial activation and innate immune responses, thereby suppressing JEV replication [177]. In addition, miR-29b and miR-301a facilitate microglial activation by modulating negative regulators of NF-κB signaling; miR-29b targets TNFAIP3, and miR-301a inhibits NKRF, leading to the activation of NF-κB activity and enhanced JEV-induced inflammatory responses [178, 179]. Let-7a/b enhances TNF-α production in microglia by interacting with TLR7 and NOTCH signaling, and exosomes overloading Let-7a/b can induce neuronal death through caspase activation [180]. miRNAs also modulate astrocyte-mediated neuroinflammation in JEV infection; miR-19b-3p and miR-15b are upregulated in JEV-infected astrocytes, both exacerbating JEV-induced inflammatory responses via the negative regulation of RNF11 and RNF125, respectively [173, 181].

Infection with human immunodeficiency virus type 1 (HIV-1) leads to the progressive weakening of the host's immune system and causes acquired immunodeficiency syndrome (AIDS). HIV-1 infection is often associated with chronic brain inflammation, and approximately 70% of patients develop HIV-1-associated neurological disorders (HAND) [182]. HIV can infect the resident cells of the CNS, including microglia and astrocytes, resulting in severe neuroinflammatory consequences [183]. HIV-1 transactivator of transcription (Tat) is an HIV-1-encoded viral protein that has attracted considerable attention because of its toxicity to CNS cells. HIV-1 Tat influences the functional dynamics of microglia and that miRNAs are important regulators of this process. Exposure of microglia to Tat leads to increased miR-34a expression, which targets the NF-κB negative regulator, NLRC5, and promotes microglial activation [184]. Tat decreases miR-124 expression by mediating DNA methylation of the miR-124 promoter, resulting in the upregulation of STAT3 and subsequent microglial activation [185]. miR-146a levels increase in HIV-1-infected primary human fetal microglia, and MCP-2 has been identified as a target of miR-146a [186]. miRNAs can modulate oxidative stress in microglia upon Tat treatment; exposure of microglia to Tat downregulates the level of miR-17, which in turn increases the expression of NOX2 and NOX4, thereby promoting ROS generation [182]. Tat upregulates the expression of miR-505 in microglia. miR-505 contributes to the production of mitochondrial superoxide and microglial senescence-like phenotypes by negatively regulating SIRT3 expression [187]. In addition, HIV-1-latently infected astrocytes can express and secrete Tat, which induces pathological and neurobehavioral changes in the CNS. In addition, Tat can cause astrocyte-mediated neuronal neurotoxicity via the miR-320a/VDAC1 axis, and overexpression of miR-320a inhibits Tat-mediated ATP release and prevents neuronal death [188]. miR-132 expression is induced by Tat through CREB phosphorylation in astrocytes, and the uptake of exosome-delivered miR-132 into neurons induces the downregulation of BDNF and MECP2, which impairs neurite outgrowth and neuronal survival [189]. Similarly, miR-29b is present in astrocyte-derived exosomes and is taken up by neurons, leading to attenuated PDGF-B expression and subsequent neuronal dysfunction [190]. In addition, miR-155 and miR-181a have been implicated in HIV-1 replication. The overexpression of these two miRNAs promotes HIV-1 replication in astrocytes by modulating SAMHD1 [191]. In response to HIV-1 infection, lncHEAL expression is upregulated in microglia, macrophages, and T lymphocytes, facilitating HIV-1 replication by interacting with the RNA-binding protein, FUS [192]. In addition, lncBACE1-AS has been implicated in Tat-mediated astrocytic amyloidosis. It binds to HIF-1α, forming the BACE1/lncBACE1-AS RNA duplex and increasing BACE1 protein and astrocytic amyloidosis [193] (Fig. 1, Table 3)

**Table 3:** ncRNAs in the regulation of astrocyte- and microglia-mediated neuroinflammation in CNS infectious diseases

	ncRNAs	Target	Function
JEV	miR-146a↑		Inhibits JEV-induced neuroinflammation [174]
JEV	miR-146a↑	TRAF6, IRAK1, IRAK2, STAT1	Facilitates JEV replication [175]
JEV	miR-155↑	SHIP1	Promotes JEV-induced neuroinflammation [176]
JEV	miR-155↑		Suppresses JEV virus replication [177]
JEV	miR-29b↑	TNFAIP3	Governs microglia activation [178]
JEV	miR-301a↑	NKRF	Promotes JEV-mediated neuroinflammation [179]
JEV	let-7a/b↑		Induces TNFα production and facilitates neuronal death [180]
JEV	miR-19b-3p↑	RNF11	Positively regulates the JEV-induced inflammatory response [181]
JEV	miR-15b↑	RNF125	Promotes JEV-induced neuroinflammation [173]
HIV-1	miR-34a↑	NLRC5	Promotes microglial activation inflammation [184]
HIV-1	miR-124↓	MECP2, STAT3	Promotes microglial activation [185]
HIV-1	miR-146a↑	MCP-2	Maintains HIV-mediated chronic inflammation [186]
HIV-1	miR-17↓	NOX2, NOX4	Suppresses ROS production in microglia [182]
HIV-1	miR-505↑	SIRT3	Facilitates mitochondrial oxidative stress [187]
HIV-1	miR-320a↓	VDAC1	Increases astrocyte-mediated neurotoxicity [188]
HIV-1	miR-132↑	MecP2, BDNF	Impairs neurite outgrowth and neuron survival [189]
HIV-1	miR-29b↑	PDGFB	Exerts neurotoxic effects on neurons [190]
HIV-1	miR-155/181a	SAMHD1	Increases HIV-1 replication in astrocytes [191]
HIV-1	lncHEAL↑	FUS	Facilitates HIV-1 replication [192]
HIV-1	lncBACE1-AS↑	BACE1	Promotes Tat-mediated astrocytic amyloidosis [193]
*E. coli*	miR-155↑	TAB2, EGFR	Suppresses *E. coli*-induced neuroinflammatory responses [201]
*E. coli*	miR-146a↑	IRAK1, TRAF6, EGFR	Suppresses *E. coli*-induced neuroinflammatory responses [201]
*L. mo*	miR-155↑	TAB2	Regulates brain inflammation [204]
*C. neoformans*	miR-30c-5p↓	eIF2α	Inhibits inflammatory responses and promotes microglia survival [205]
*M. tb*	miR-124↓	STAT3	Promotes microglia apoptosis and the elimination of *M. tb* [206]
*S. pneumoniae*	miR-141-3p↓	HMGB1	Inhibits the activation of astrocytes and inflammatory responses [207]
*S. pneumoniae*	miR-135a↓	HIF-1α	Facilitates proliferation and inhibits apoptosis of astrocytes [208]

.

### Bacterial encephalitis

Bacterial infection of the CNS is a significant global public health problem with high mortality and morbidity rates [194]. Based on the affected anatomical regions, CNS infections can be classified as encephalitis, meningitis, or myelitis, with bacterial meningitis being the most common and severe disease [195]. *Escherichia coli* (*E. coli*), *Streptococcus pneumoniae* (*S. pneumoniae*), *Listeria monocytogenes* (*L. mo*), *Mycobacterium tuberculosis* (*M. tb*), *Neisseria meningitidis,* and *Haemophilus influenzae type b* are the major causes of meningitis [196, 197]. Bacteria multiply in the subarachnoid space and release compounds related to pathogen-associated molecular patterns (PAMPs), such as lipopolysaccharides, peptidoglycan, flagellin, DNA, and lipoteichoic acid. Microglia and astrocytes can induce immune responses by recognizing PAMPs through pattern recognition receptors, thereby facilitating the elimination of invasive bacteria [198]. However, an excessive inflammatory response can cause neuronal damage and death. Therefore, the intensity and duration of inflammatory responses should be maintained at appropriate levels, and miRNAs are critical regulators of glia-mediated neuroinflammation during bacterial infections [199].

Our group has previously profiled the expression of miRNAs in *E. coli*-infected astrocytes [200]. Transcriptome data showed that 16 miRNAs were upregulated and 11 miRNAs were downregulated compared to the control group. We further characterized the roles of the most significantly upregulated miRNAs, miR-155 and miR-146a [201]. These two miRNAs collectively attenuate *E. coli*-induced neuroinflammatory responses through negative feedback regulation of the TLR-mediated NF-κB and EGFR/NF-κB signaling pathways. Lipopolysaccharide (LPS), a major component of several Gram-negative bacteria, is known to be an important endogenous promoter of neuroinflammation. Studies suggest that low expression of miR-138-5p after LPS administration may contribute to the activation of NLRP3/caspase-1 in microglia, leading to hippocampal neuroinflammation [202, 203]. In addition, miR-155 modulated brain inflammation via multiple mechanisms during *L. mo* infection. Peripheral miR-155 contributes to inflammation by promoting the recruitment of T lymphocytes, whereas microglial miR-155 plays a dual role: miR-155 can enhance M1 microglial polarization and suppress inflammatory responses by targeting TAB2 [204]. miR-30c-5p is beneficial during *Cryptococcus neoformans* infection as it attenuates the release of inflammatory cytokines and promotes microglial survival [205]. Since *M. tb* is internalized and replicates within microglia, miR-124 is thought to protect against *M. tb* infection by promoting microglial apoptosis and facilitating *M. tb* clearance. Mechanistically, miR-124 directly targets STAT3 and abrogates the anti-apoptotic effects of STAT3 signaling [206]. In an *S. pneumoniae*-induced bacterial meningitis model, miR-141-3p expression decreased in the brain tissue and astrocytes. Overexpression of miR-141-3p suppresses astrocyte activation and inflammatory responses by negatively regulating HMGB1 [207]. miR-135a is also downregulated in brain tissue of *S. pneumoniae*-infected rats, facilitating proliferation and inhibiting apoptosis of astrocytes by targeting HIF-1α [208] (Fig. 1, Table 3).

## Conclusion and future directions

Astrocytes and microglia, the most important components of the innate immune system in the CNS, constantly monitor the brain microenvironment under normal conditions. They can be activated following inflammation, infection, or trauma and play an important role in the pathological process and development of CNS diseases. The roles of miRNAs, lncRNAs, and circRNAs in regulating astrocyte- and microglia-mediated neuroinflammation during CNS diseases are summarized in this review. It is well-known that ncRNAs regulate the expression of genes involved in various signaling pathways in astrocytes and microglia that contribute to neuroinflammation. Disruption of these signaling cascades can be achieved by the overexpression or inhibition of ncRNAs, allowing astrocytes and microglia to revert to the neuroprotective phenotype involved in CNS repair and recovery.

**Fig. 2:**
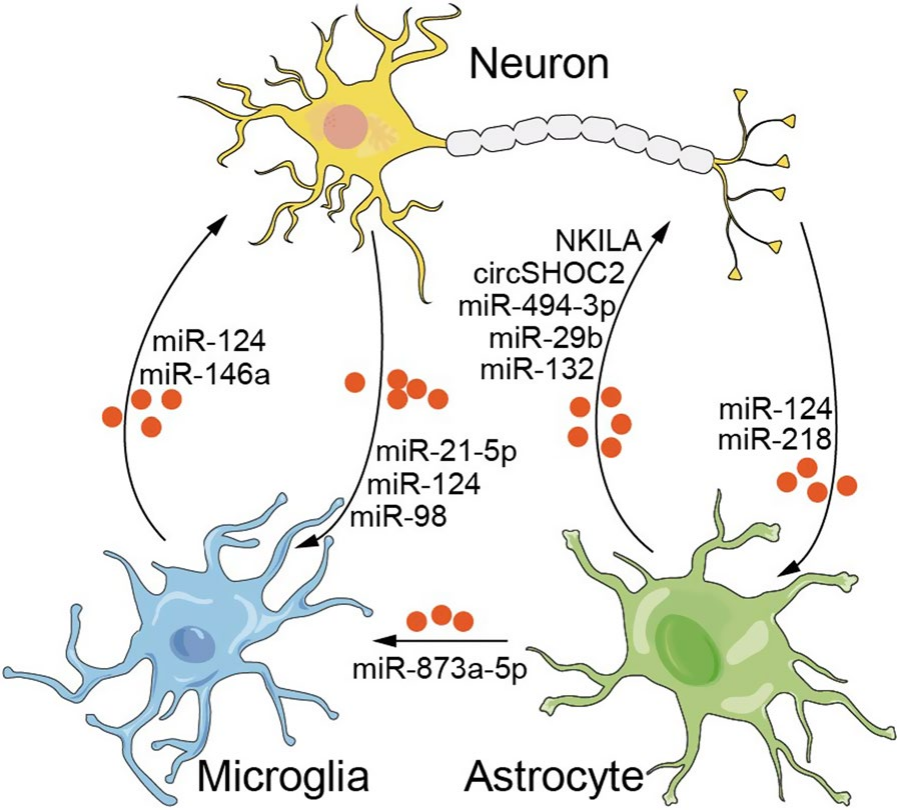
Brief summary of ncRNAs crosstalking between neurons, astrocytes, and microglia in CNS diseases

Different types of brain cells work together to maintain healthy brain activity. Under normal physiological conditions, neurons and glia communicate with and integrate signals from the surrounding cells and the environment to maintain CNS stability. Disturbances in the neuron–glia crosstalk contribute to various pathological states of CNS diseases. Under pathological conditions, the release, recognition, and uptake of molecules or other cellular components by neurons, astrocytes, and microglia are altered. Therefore, a better understanding of the crosstalk between neurons, astrocytes, and microglia is essential to understand the mechanisms of neuroinflammation in CNS diseases. This review summarizes the ncRNAs involved in intercellular communication in CNS diseases (Fig. 2). In particular, ncRNA research has opened new therapeutic strategies for the treatment of neuroinflammation, because ncRNAs can be packaged as exosomes, which are better able to cross the BBB [209]. For instance, in AD, treatment with miR-22-loaded exosomes significantly inhibited M1 microglial differentiation and the expression of inflammatory factors [28]. In IS, miR-146a-loaded exosomes inhibit microglial activation and neuroinflammation via the IRAK1/TRAF6 pathway [93]. Therefore, the development of therapies using ncRNA cocktails and exosome gene therapy has great potential and opens new avenues for personalized treatment of neurological diseases.

## Data Availability

Not applicable.

## Abbreviations

CNS: Central nervous system
BBB: Blood–brain barrier
GFAP: Glial fibrillary acidic protein
ncRNAs: Non-coding RNAs
miRNA: MicroRNA
lncRNA: Long non-coding RNA
circRNA: Circular RNA
UTRs: Untranslated regions
nt: Nucleotides
mRNA: Messenger RNA
AD: Alzheimer’s disease
TLR7: Toll-like receptor 7
Aβ: Amyloid β
IGF1: Insulin-like growth factor 1
TREM2: Triggering receptor expressed on myeloid cells-2
MEG3: Maternally expressed gene 3
NSC: Neural stem cells
UCA1: Urothelial carcinoma-associated 1
AP2B1: Adaptor protein 2 B1
PD: Parkinson’s disease
MPTP: 1-Methyl-4-phenyl-1,2,3,6-tetra hydropyridine
PTEN: Phosphatase and tensin homolog
NLRP3: Nod-like receptor protein 3
SNHG1: Small nucleolar RNA host gene 1
ceRNA: Competing endogenous RNA
EZH2: Enhancer of zeste homologue 2
NRF2: Nuclear factor-like-2 factor
FSTL1: Follistatin-like 1
ALS: Amyotrophic lateral sclerosis
MN: Motor neurons
SOD1: Superoxide dismutase 1
mSOD: Symptomatic SOD1
ADEV: Astrocyte-derived extracellular vesicles
MS: Multiple sclerosis
EAE: Experimental autoimmune encephalomyelitis
OPC: Oligodendrocyte progenitor cell
EVs: Extracellular vesicles
IL-17: Interleukin-17
GAS5: Growth arrest-specific 5
SF: Sulfasalazine
IS: Ischemic stroke
I/R: Ischemia–reperfusion
OGD: Oxygen–glucose deprivation
MCAO: Middle cerebral artery occlusion
TBI: Traumatic brain injury
SCI: Spinal cord injury
DHA: Docosahexaenoic acid
MSC: Mesenchymal stem cell
JEV: Japanese encephalitis virus
JE: Japanese encephalitis
HIV-1: Human immunodeficiency virus type 1
Tat: HIV-1 transactivator of transcription
*E. coli*: *Escherichia coli*
*S. pneumoniae*: *Streptococcus* *pneumoniae*
*L. mo*: *Listeria monocytogenes*
*M. tb*: *Mycobacterium tuberculosis*
PAMPs: Pathogen-associated molecular patterns
LPS: Lipopolysaccharide
